# First Insight into the Kinome of Human Regulatory T Cells

**DOI:** 10.1371/journal.pone.0040896

**Published:** 2012-07-16

**Authors:** Sebastian König, Michael Probst-Kepper, Tobias Reinl, Andreas Jeron, Jochen Huehn, Burkhart Schraven, Lothar Jänsch

**Affiliations:** 1 Department of Molecular Structural Biology, Helmholtz-Zentrum für Infektionsforschung, Braunschweig, Germany; 2 Institute for Microbiology, Immunology and Hygiene, Städtisches Klinikum Braunschweig gGmbH, Braunschweig, Germany; 3 Department of Experimental Immunology, Helmholtz-Zentrum für Infektionsforschung, Germany; 4 Institute of Molecular and Clinical Immunology, Otto-von-Guericke Universität, Magdeburg, Germany; New York University, United States of America

## Abstract

Regulatory T cells (Tregs) are essential for controlling peripheral tolerance by the active suppression of various immune cells including conventional T effector cells (Teffs). Downstream of the T cell receptor (TCR), more than 500 protein kinases encoded by the human genome have to be considered in signaling cascades regulating the activation of Tregs and Teffs, respectively. Following TCR engagement, Tregs posses a number of unique attributes, such as constitutive expression of Foxp3, hyporesponsiveness and poor cytokine production. Furthermore, recent studies showed that altered regulation of protein kinases is important for Treg function. These data indicate that signaling pathways in Tregs are distinctly organized and alterations at the level of protein kinases contribute to the unique Treg phenotype. However, kinase-based signaling networks in Tregs are poorly understood and necessitate further systematic characterization. In this study, we analyzed the differential expression of kinases in Tregs and Teffs by using a kinase-selective proteome strategy. In total, we revealed quantitative information on 185 kinases expressed in the human CD4^+^ T cell subsets. The majority of kinases was equally abundant in both T cell subsets, but 11 kinases were differentially expressed in Tregs. Most strikingly, Tregs showed an altered expression of cell cycle kinases including CDK6. Quantitative proteomics generates first comparative insight into the kinase complements of the CD4^+^ Teff and Treg subset. Treg-specific expression pattern of 11 protein kinases substantiate the current opinion that TCR-mediated signaling cascades are altered in Tregs and further suggests that Tregs exhibit significant specificities in cell-cycle control and progression.

## Introduction

Regulatory T cells (Tregs) essentially contribute to immune homeostasis by the active suppression of conventional T effector cells (Teffs). The absence or dysfunction of Tregs can lead to severe autoimmune diseases, while increased Treg numbers can prevent efficient immune responses against tumors or invading pathogens [1], [2], [3], [4]. It is believed that T cells with regulatory function exist in all major T cell subsets. Most studies have focused on Tregs of the CD4^+^ lineage constitutively expressing high levels of the IL-2 receptor α-chain (CD25) and the transcription factor “forkhead box P3” (Foxp3), which is essential for the Treg development and function [5]. Although it has been established that active transcription of Foxp3 requires signals from the antigen-specific T-cell receptor (TCR) and other co-stimulatory molecules [6], [7], [8], [9], [10], such as CD28, our knowledge about intracellular signaling cascades controlling Foxp3 expression and Treg function is still limited.

In contrast, TCR signaling in conventional Teffs has been studied intensively over the last decades. Following TCR engagement, protein kinases coordinate virtually all intracellular signaling pathways by introducing phosphorylations on serines, threonines and tyrosines of their protein substrates. TCR stimulation induces the activation of tyrosine kinases of the SRC, SYK and TEC family, which leads to the phosphorylation the adaptor protein “linker of activated T cells” (LAT) [11], [12]. The recruitment of various effector proteins to phosphorylated LAT leads to the formation of a signaling module controlling the activation of downstream signaling cascades, such as the “phosphatidylinositol 3-kinase” (PI3K), “protein kinase C” (PKC) and “mitogen-activated protein kinase” (MAPK) pathway. These signaling cascades regulate the expression of transcription factors, cytoskeleton rearrangements, cell cycle progression, cytokine production and the induction of T cell effector functions.

Notably, Tregs possess a number of unique attributes and intracellular signaling cascades that differ from those of conventional Teffs. For example, Tregs possess properties of T cell anergy, are hyporesponsive, show a poor cytokine production and actively suppress other immune cells following TCR activation [13], [14], [15], [16]. Studies from different laboratories suggest that signaling cascades downstream of the TCR and IL-2 receptor are distinctly organized in Tregs [14], [15], [17], [18]. Moreover, there is recent evidence that altered activities of AKT (Protein Kinase B), glycogen synthase kinase 3-beta (GSK3B) and protein kinase C-theta (PKC-theta) contribute to the suppressive capacity of Tregs [9], [19], [20], [21]. These data indicate that certain signaling pathways in Tregs are distinctly organized and that altered kinase regulation is important for Treg function. However, kinase-based signaling networks in Tregs were not characterized systematically and are poorly understood at the present. In particular, the comparison of kinase expression in Tregs and their Teff counterparts might reveal Treg-specific network alterations underlying the Tregs’ unique phenotype.

More than 500 different protein kinases encoded by the human genome (kinome) [22] have to be considered in intracellular signaling cascades of Tregs. Differential gene expression in Tregs and Teffs has been studied systematically over the last years using array technologies [23], [24], [25], [26], [27], [28] allowing the analysis of virtually all genes. However, results regarding kinases are rarely presented and discussed that likely is caused by low mRNA levels favoring the idea to investigate these signaling molecules preferentially at the protein level. On the other hand, comparative proteome analysis necessitates substantial cell amounts and thus was hindered by the scarcity of CD4^+^CD25^high^Foxp3^+^ Tregs and their poor proliferation *in vitro*. Nevertheless, two proteome studies have compared protein expression in human Tregs and Teffs isolated from leukapheresis and buffy coat products [29], [30]. In total, these studies have identified Galectin-10 and CD147 (Basigin/Emmprin) as Treg-specific marker proteins, but provided no information about low abundant protein kinases at all. Recent advances in chemical proteomics already demonstrated that kinase-selective mass spectrometry (kinomics) could be used to comprehensively characterize kinase networks of eukaryotic cells [31], [32], [33], [34], [35], [36]. Here, we combined *ex vivo* T cell expansion allowing the generation of high numbers of *bona fide* Treg and Teff fractions matching the criteria of reproducibility and required cell amounts for a kinase-selective proteome study. This kinome approach gained insight into a substantial portion of the human CD4^+^ T cell kinome and moreover revealed individual kinases, which showed a Treg-specific expression coinciding with the unique Treg phenotype.

## Results

### Characterization of *ex vivo* Expanded Human CD4^+^ Tregs and Teffs

**Figure 1:**
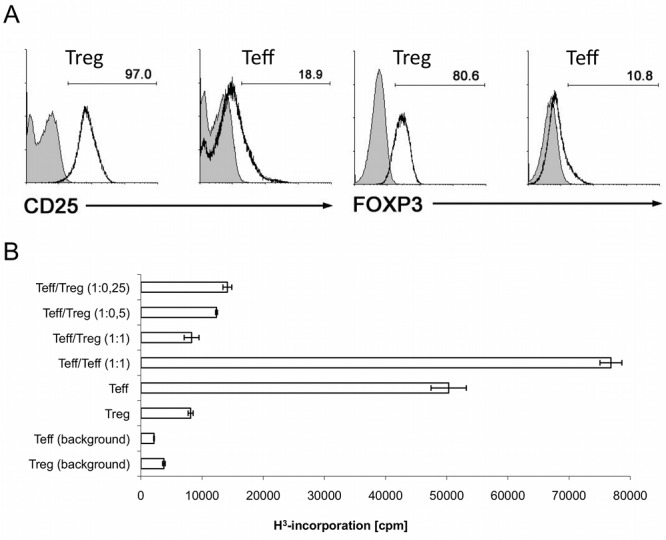
Phenotype and function of *ex vivo* expanded Tregs and Teffs. (**A**) Allo-reactive CD4^+^CD25^hi^ Tregs expressed high levels of CD25 (95–99%) and Foxp3 (80–90%). Allo-reactive CD4^+^CD25^−^ Teffs showed low levels of CD25 and Foxp3 expression. (**B**) T cell proliferation and suppressive capacity of Tregs was accessed by ^3^H (thymidine)-incorporation on day 3 in triplicates prior each kinome analysis. Expanded Tregs consistently suppressed the proliferation of corresponding Teffs. Data shown are representative for Teffs and Tregs used in this study.

Although the sensitivity of mass spectrometry (MS) devices has significantly increased over the past ten years, the characterization of low abundant signaling molecules such as protein kinases still requires substantial cell numbers (>10^8^). Since CD25^high^ Tregs comprise only 5–10% of the total CD4^+^ T cell population [37], we here used a previously described, alloantigen-driven *ex vivo* expansion system to generate sufficient numbers (up to 2×10^8^) of human CD25^−^CD4^+^ Teffs and CD25^high^CD4^+^ Tregs [23], [25]. Importantly, expanded alloantigen-specific Tregs showed high levels of CD25 and FOXP3 expression, whereas expanded alloantigen-specific Teffs expressed only low levels of CD25 and FOXP3 (Figure 1a). In contrast to Teffs, expanded Tregs showed an anergic phenotype and, additionally, efficiently suppressed the proliferation of alloantigen-specific Teffs (Figure 1b). Thus, essential phenotypical and functional Treg characteristics were maintained during their *in vitro* expansion.

**Figure 2:**
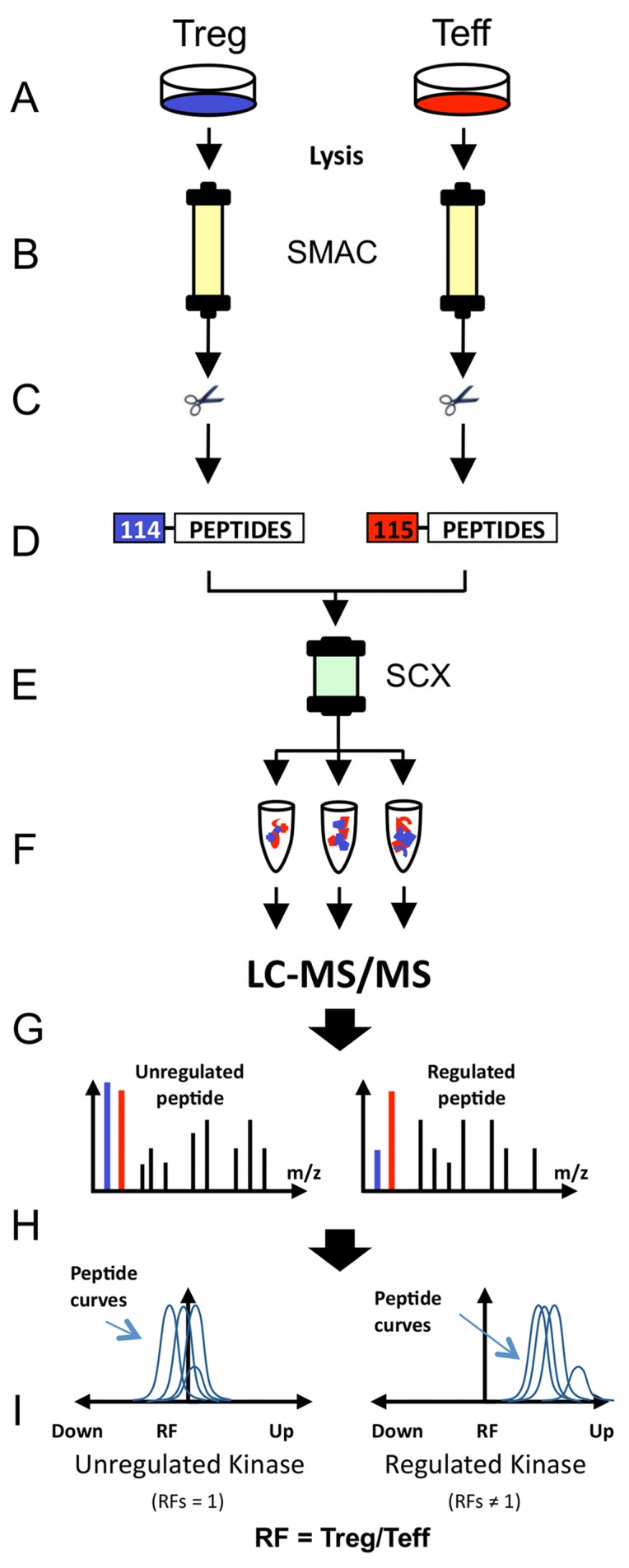
Experimental strategy for the comparative analysis of kinase-dependent signaling networks in Tregs and Teffs. (**A**) *Ex vivo* expansion of human T cell subsets. (**B**) Protein kinase purification by VI16743/Purvalanol-B-based small molecule affinity chromatography (SMAC). (**C**) Kinase elution, denaturation and tryptic digestion. (**D**) Quantitative peptide labeling with iTRAQ™ and sample combination. (**E**) Separation of complex peptide sample by Strong Cation Exchange Chromatography (SCX). (**F**) LC-MS/MS analysis of multi-dimensional separated peptide fractions. (**G**) Mascot database search and peptide/kinase identification. (**H**) Statistical evaluation of quantitative MS data (iTRAQassist). (**I**) Visualization of expression data. Regulation factors (RFs) describe the relative kinase expression level in Tregs vs. Teffs (RF = Treg/Teff).

### The Kinase Complement of Human CD4^+^ Tregs and Teffs

**Figure 3:**
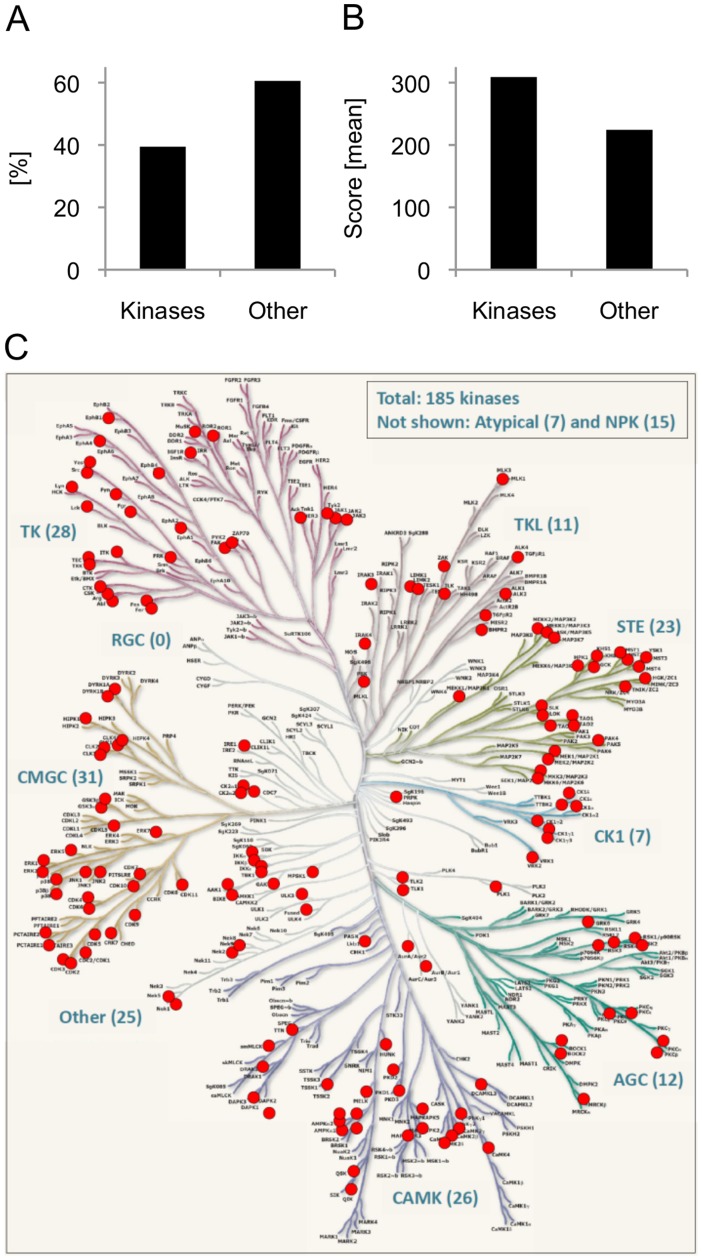
Kinase-selective proteomics provided comprehensive insight into the kinase complement of human Tregs and Teffs. (**A**) Enrichment of kinases. Percentage of kinases and non-kinases (others: proteins without kinase activity) after VI16743/Purvalanol-B-mediated affinity purification is shown. (**B**) Quality of kinase identification. Mean Mascot Mowse score of kinases *vs*. non-kinases (others). The Mascot score is reflecting the probability of the match between the mass spectrometry spectra/data and the protein sequence given in the database. Data shown in A and B are representative for four independently performed LC-MS/MS experiments. (**C**) Kinase complement of human Tregs and Teffs. In total, 185 kinases were identified. Kinases from nearly all groups of the human kinome were detected: AGC, PKA/PKG/PKC-family kinases; CAMK, calcium/calmodulin-dependent kinases; CK1, casein kinases; CMGC, CDK/MAPK/GSK3/CLK-family kinases; RCG, receptor guanylate cyclases; STE, sterile homologue kinases; TK, tyrosine kinases; TKL, tyrosine kinase-like kinases; atypical protein kinases; Other, kinases belonging to non of the mentioned groups; NPK, non-protein-kinases (kinases having non protein substrates). The kinase dendrogram was adapted with permission from Cell Signaling Technology, Inc. (www.cellsignal.com).

More than 500 distinct protein kinases are encoded by the human genome (kinome) [22] and constitute the framework of almost all signaling pathways. We here applied a chemical proteome strategy that is based on an efficient pre-enrichment of kinases followed by biochemical and mass spectrometric methods to comprehensively and comparatively analyze the kinome of human CD4^+^ Tregs and Teffs, respectively (Figure 2). The kinase pre-enrichment was achieved by the use of highly unspecific ATP-competitive kinase inhibitors VI16743 and Purvalanol-B. VI16743 was derived from the pyridopyrimidine class inhibitor PP58 by removing it’s dichlorophenyl side chain and replacing the methyl group of PP58 by a cyclopentyl ring resulting in a small molecule permitting comprehensive access to kinases constituting part in major signal transduction pathways such as mitogen-activated kinase (MAPK) signaling [31]. The inhibitory spectrum of VI16743 was complemented by the commercially available inhibitor Purvalanol-B, particularly targeting cell cycle-associated kinases controlling signal integration and cell cycle progression [33], [38], [39]. Using a serial combination of immobilized kinase inhibitors VI16743 and Purvalanol-B, kinases were directly enriched from total T cell lysates by two consecutive small molecule affinity chromatography steps (SMAC) (Figure 2). Approximately 40% of the proteins within the SMAC eluate fractions were classified as kinases (Figure 3a), and these molecules were identified with a significant higher mean Mascot score compared to other proteins (Figure 3b), reflecting a better peptide identification and coverage of kinases achieved by mass spectrometry. These data clearly underscore the high kinase selectivity of the used kinase inhibitors VI16743 and Purvalanol-B. Repeated generation of lysates from >2×10^8^ human Tregs and Teffs provided enough material for four comprehensive kinome experiments. In total, the expression of 185 kinases was detected in human Tregs and Teffs (Supplementary Table S1). Among these, the majority (n = 170) constituted protein kinases and only 15 used low molecular weight phosphate acceptors as substrates (non-protein kinases, NPKs). The assignment of identified protein kinases to the human kinome dendrogram [22] demonstrated that kinases virtually from all branches of the human kinome have been accessed by this proteome approach (Figure 3c). Altogether, this work for the first time provided insight into a significant portion of the kinome of human CD4^+^ Tregs and Teffs, particularly accessing kinases being essential for signal transduction, signal integration and cell-cycle control.

### Differential Kinase Expression in Human Tregs and Teffs

**Figure 4:**
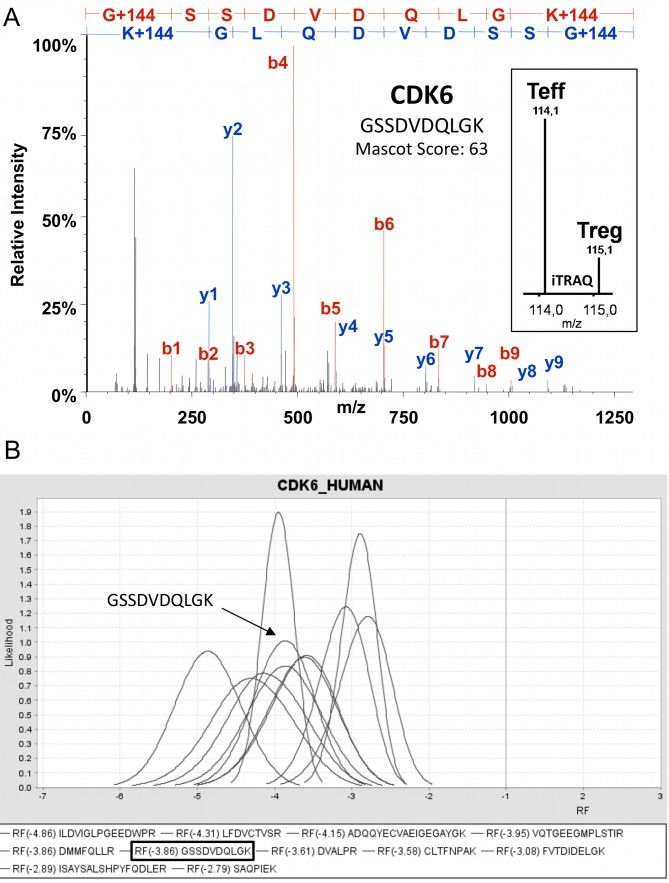
iTRAQ-based quantification and statistical evaluation of relative kinase expression in Tregs and Teffs. iTRAQ-based quantification and statistical analysis of differential kinase expression in Tregs and Teffs is exemplarily shown using the example of cyclin-dependent kinase 6 (CDK6). (**A**) MS/MS fragmentation spectrum of a tryptic peptide derived from cyclin-dependent kinase 6 (CDK6). Ions used for peptide sequencing and protein identification are indicated in red (b ions series) or blue (y ions series). The resulting peptide sequence (GSSDVDQLGK) is shown in the upper part of the diagram. The box embedded in the graph displays a magnification of the low molecular mass range where the iTRAQ reporter intensities could be observed. The intensity of the iTRAQ reporter 115 is correlating with the peptide abundance in Tregs. The peak at 114 Da represents the amount of the respective peptide in Teffs. (**B**) Statistical analysis of relative CDK6 peptide abundances. Analyses were performed by iTRAQassist as described previously [40]. Most likely (see also Table 1) and further possible peptide regulations were calculated and depicted as likelihood curves for every peptide. All peptides derived from CDK6 were significantly less abundant in Tregs. All peptide regulations calculated for individual CDK6 peptides were used to determine the overall regulation of the CDK6 protein (see Table 1). Here CDK6 peptide data are shown obtained from one representative LC-MS/MS experiments.

To characterize the abundance of kinases at the protein level comparatively in Tregs and Teffs, peptides derived from proteins enriched by SMAC were differentially labeled with isobaric iTRAQ molecules, allowing their parallel identification and precise relative quantification by mass spectrometry (MS). Labeled peptides were separated by reverse phase chromatography and sequenced by tandem MS (LC-MS/MS). Furthermore, we used SCX chromatography as a second dimension to prefractionate and support the systematic analyses of kinase peptides. All peptide fractions were measured separately, resulting MS raw data were compiled and protein identification and quantification was performed at the level of single MS/MS peptide spectra as exemplified by a fragment ion spectra derived from the cyclin-dependent kinase 6 (CDK6) (Figure 4a). The peptide with the amino acid sequence GSSDVDQLGK was exclusively assigned to CDK6 and intensities of iTRAQ reporters released from the peptide during the fragmentation process were used for relative peptide quantification (Figure 4a, box). The presented CDK6 peptide was obviously less abundant in Tregs. However, regulatory information can basically vary in robustness according to the signal-to-noise characteristics of the MS devise. Therefore, we used a novel and stringent statistical bioinformatics approach iTRAQassist [40], which allowed the unambiguous detection of differential protein expression at the single peptide level in all cases. Computed regulation values were depicted as a function of their likelihood for every unique peptide, as shown exemplarily for CDK6 peptides from representative LC-MS/MS experiments (Figure 4b). The corresponding CDK6 peptide regulation factors are given in Table 1. All CDK6 peptide regulation curves were clearly separated from the y-axis (RF = 1) underscoring the statistical significance of the observed CDK6 regulation. For this reason, cumulative regulation factors for every protein kinase were determined based on quantitative information of all unique peptides belonging to the respective kinase. Data belonging to CDK6 indicate that this cell cycle kinase is at least three-times less abundant at the protein level in human CD4^+^ Tregs in comparison to Teffs.

**Table 1:** Differential CDK6 peptide abundances in Tregs and Teffs.

Name [UniProt]	Peptide sequence	Mascot score	Relative abundance^1^ [RF = Treg/Teff]
CDK6	ADQQYECVAEIGEGAYGK	50	−4,2
CDK6	CLTFNPAK	44	−3,6
CDK6	DMMFQLLR	28	−3,9
CDK6	DVALPR	30	−3,6
CDK6	FVTDIDELGK	70	−3,1
CDK6	FVTDIDELGKDLLLK	43	−3,1
CDK6	GSSDVDQLGK	63	−3,9
CDK6	ILDVIGLPGEEDWPR	77	−4,9
CDK6	ISAYSALSHPYFQDLER	50	−2,9
CDK6	LFDVCTVSR	38	−4,3
CDK6	SAQPIEK	30	−2,8
CDK6	VQTGEEGMPLSTIR	86	−4,0
CDK6	Total protein	2018	^§^−3,7

iTRAQ-based quantification in combination with a MS-devise-specific statistical approach (iTRAQassist) [40] was used to determine relative peptide abundances^1^. Peptide regulations were used to calculate the overall regulation^§^ of the respective kinase. Here, exemplarily CDK6 peptide data are shown obtained from one representative experiments.

**Figure 5:**
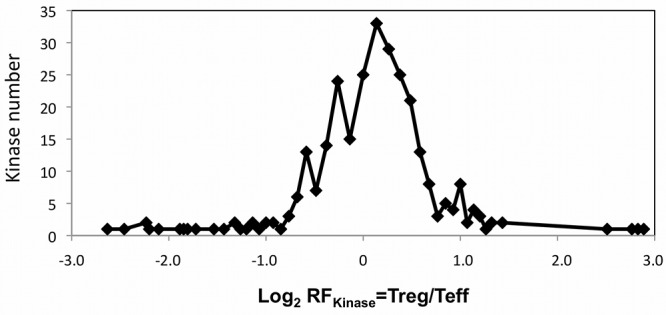
Distribution of iTRAQ-based kinase regulation in Tregs and Teffs. Here the log2 values of kinase expression ratios in Tregs and Teffs overall conducted LC-MS/MS experiments are shown (log2 RF_kinase_ = Treg/Teff). The majority of kinases was equally expressed in both T cell subsets.

In total, this study revealed robust quantitative information on 170 protein and 15 non-protein kinases expressed in human CD4^+^ T cell subsets. Considering by-product corrections and signal-to-noise characteristics of iTRAQ reporter intensities, none of the identified kinases was exclusively expressed in either Tregs or Teffs. In fact, the majority of kinases was equally abundant in both T cell subsets as demonstrated by cluster analysis showing a normal distribution of kinase regulation factors over all conducted kinome experiments (Figure 5). However, quantitative data of eleven protein kinases revealed their differential expression in the investigated Tregs and Teffs (Table 2 and Supplementary Table S1). The expression of calcium/calmodulin-dependent protein kinase II delta (KCC2D), a component of the canonical TCR signaling network [41], [42], [43], was increased in Tregs, whereas the proto-oncogene tyrosine-protein kinase Src (SRC, alias c-SRC), so far not described in T cell activation [44], was significantly less abundant in the Treg subset when compared to Teffs. Furthermore, we observed an altered expression of aurora kinase B (AURKB) and serine/threonine-protein kinase 10 (STK10, alias LOK), both components of the CD28-dependent co-stimulatory pathway. AURKB, acting as a positive effector of CD28-dependent T cell activation [45], [46], [47], [48], was significantly down regulated in Tregs, whereas STK10, which negatively influences CD28 signaling [49], was slightly increased. STK4 (alias MST1), which together with STK10 is involved in LFA-1-(lymphocyte function-associated antigen 1)-mediated T cell adhesion [50], [51], showed also elevated expression levels in Tregs. Moreover, ephrin receptor B1 (EPHB1) *inter alia* mediating T-cell-to-T-cell interaction [52], [53], [54], [55], [56], [57], [58] was clearly up regulated in Tregs. Most significantly, Tregs showed an altered expression of cell cycle-regulating kinases controlling all cell cycle stages, i.e. NEK9, AURKB, CDK1, CDK2, CDK3 and as aforementioned CDK6 (Table 2). In total, the Treg-specific expression of all 11 protein kinases have now to be discussed and evaluated regarding their functional role in known and incompletely characterized *in vivo* phenotypes of human regulatory T cell subsets.

**Table 2:** Differential kinase abundances in Tregs and Teffs.

Name [UniProt]	Differential expression^1^ [RF_median_]	Function
Down regulated in Tregs:		
CDK1	−3,6	Cell cycle (G2, Mitosis)
AURKB	−3,6	CD28 signaling, cell cycle (S phase entry, mitosis)
CDK6	−3,2	TCR/CD28 signal integration,cell cycle (G0/G1 exit), T cell proliferation
SRC	−2,5	Not known
CDK2	−1,6	Cell cycle (S phase entry/progression)
Up regulated in Tregs:		
NEK9	1,6	Cell cycle (interphase progression and mitosis)
STK10	1,7	CD28 signaling, LFA-1-mediated adhesion
KCC2D	1,7	TCR, calcium and NFkB signaling
CDK3	2,0	Cell cycle (G0/G1 exit, G1/S transition)
STK4	2,3	LFA-1-mediated adhesion, proliferation
EPHB1	6,9	T cell co-stimulation, development, interaction, migration

Kinases regarded as expressed differentially in both T cell subsets (RF = Treg/Teff) had to met two criteria: −1.5>RF>1.5 at least in two of the conducted experiments and −1.5>RF_[median]_ >1.5 (^1^median RF over all performed experiments).

## Discussion

### Proteome Analysis of Kinase-based Signaling Networks in Tregs

Low Treg numbers *in vivo* and their poor proliferative capacity *in vitro* limits the application of existing quantitative proteome strategies at the present. So far, only one proteome study comparing human Tregs and Teffs derived from leukapheresis products was published [29]. However, this 2D-gel-based MS approach was restricted to total protein level and could not provide information on the differential expression of low abundant protein kinases. Here, we combined an alloantigen-specific expansion protocol [23], [25], which allowed an effective *in-vitro* expansion of primary Tregs (>10^8^), with established concepts of a kinase-selective proteome strategy. MS-aided sequencing identifies and confirmed the expression of 185 kinases unambiguously in both T cell subsets (Table S1). Generally, genomics has suggested that a certain cell type likely is expressing not all 500 but up to 300 kinases simultaneously [59]. Hence, this study gained insight into ∼60% of the totally expressed CD4^+^ T cell kinome. Functional annotation revealed that more than 50% of the identified kinases were involved in signal transduction and/or activation processes in T cells. Many of them being decisive for signal integration, cell-cycle control and T-cell development were identified. Regarding the currently known TCR/CD28-dependent kinase signaling network [60] quantitative data for about 80% of the involved kinase complement were obtained in this study. All MS spectra used for the identification of the expressed kinases comprise also iTRAQ reporter intensities and a thorough statistical evaluation revealed the Treg-specific regulation of 11 protein kinases. Collision cell induced fragmentation experiments thereby provided accurate and reliable quantitative MS-data that were always in accordance with conventional quantitative western blot studies as we have validated for CDK2, CDK6, KCC2D, NEK9 and STK4 (Figure S1).

**Figure 6:**
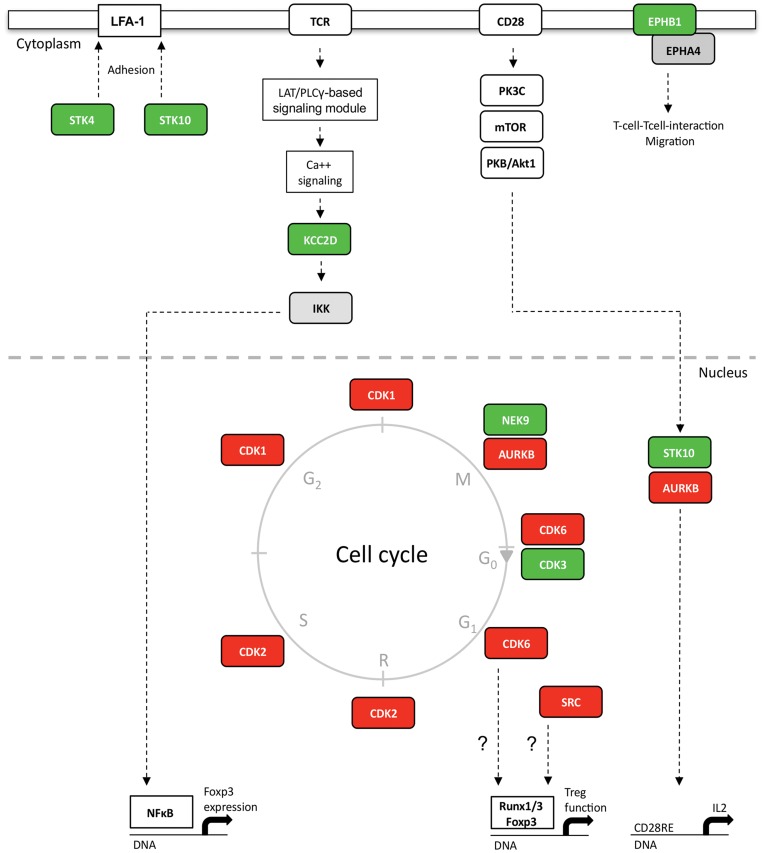
Kinase signaling network specificities of human Tregs. Comparative kinome analysis revealed differential kinase expression in Tregs and Teffs. Altered kinase expression in Tregs (RF = Treg/Teff) is indicated by green (increased expression in Tregs) and red boxes (reduced expression in Tregs). Kinases regarded as expressed differentially had to met two criteria: −1.5>RF>1.5 at least in two of the conducted experiments and −1.5>RF_[median]_ >1.5 (median RF over all performed experiments). The figure provides an overview of possible kinase functions in Tregs. Median RF values and references for indicated kinase functions are given in Table 2.

The altered protein amount of 11 out of 185 profiled kinases is now constituting a first rational basis for functional hypotheses at the proteome level about the kinase signaling network in Tregs that might not only contribute to the Foxp3-dependent suppressive phenotype, but also to unique adhesion and migration properties of Tregs (Figure 6). The increased levels of STK10 and STK4 indicate a differential regulation of the LFA-mediated T-cell adhesion [50], [51]. Whereas STK10 might also act as negative regulator of CD28-dependent IL-2 transcription [49], STK4 is additionally known to regulate lymphocyte trafficking and their interstitial motility within lymph nodes [61]. Furthermore, a functional role in integrin-mediated T-cell interaction and migration was also described for the ephrin receptor kinases EPHB1 and EPHA4 [52], [62], [63]. Here, the amount of both receptors was detected to be sevenfold increased in Tregs when compared to Teffs (Table 2 and Table S1), whereas other members of this receptor family (EPHB4, EPHA2) were equally expressed in both T cell subsets. The increased expression of calcium/calmodulin-dependent protein kinase II delta (KCC2D) in Tregs (Table 2 and Figure 6) complements our knowledge about calcium-dependent signaling [14], [17], [64]. KCC2D activates the phosphatase PP2A that, acting at Carma1, diminish the T cell activation [65]. In addition, KCC2D can promote IKK-dependent NFκB activity [41], [42], [43], which is a positive regulator of Foxp3 expression [66], [67]. Thus, an elevated KCC2D level is supporting a hypo-responsive phenotype and Foxp3-dependent functions.

The relevance of diminished AKT activity in Tregs was already demonstrated [18], [19]. AURKB, a downstream target of AKT, plays a pivotal role in mTOR-driven cell cycle progression and IL2-induced T cell proliferation and was found to be significantly reduced together with further downstream components CDK1 and CDK2 [46], [47], [48] in this study.

Beside of AURKB, CDK1 and CDK2, the differential expression of further kinases controlling the cell cycle, i.e. NEK9, CDK3 and CDK6, was detected. The alterations of all six kinases indicate a limited Treg-capability for mitosis and cell cycle-progression, but functional implications in T cells are only available for CDK1, CDK2 and mostly for CDK6. Interestingly, it is known that anergy of T cells is strongly associated with decreased CDK1/CDK2 activity which results into G_1_ cell cycle arrest [68]. Consequently, the reduced expression of kinases promoting interphase and mitotic progression might contribute to the anergic phenotype of Tregs [13], [69]. In contrast, it is already known that CDK6 contribute to the integration of TCR/CD28-derived signals [70], [71], [72], [73], [74] and is essential for the proliferation of thymic and peripheral T cells [75], [76], [77]. Here, we demonstrated that CDK6 is significantly down regulated in Tregs. Notably, CDK6 can also bind the Foxp3 interaction partner Runx1 [78], which is critically involved in both Foxp3 expression and Foxp3-dependent Treg function [79], [80], [81], [82], [83], [84], [85]. CDK6 directly bind to the runt domain of Runx1 and thereby inhibit the transcriptional activity of Runx1 [78]. Therefore, Treg-specific down-regulation of CDK6 supports the availability of Runx1 for Foxp3 interaction and the maintenance of related phenotypes. In accordance, preliminary data indicate only minor donor variations of the reduced CDK6 levels supporting the assumption of a conserved feature in human Tregs (Figure S2).

SRC kinase regulation can further support the maintenance of Runx3/Foxp3-dependent phenotypes [79], [83], [86]. Recent data provide evidence that Runx3 is directly phosphorylated by SRC and thereby is retained in the cytoplasm [87]. Thus, diminished SRC amounts as detected in the present study might preserve the nuclear localization of Runx3 and along with reduced CDK6 protein levels facilitate Foxp3 expression and thereby Foxp3-associated Treg functions.

This study gave comprehensive insights into the kinase complement of human CD4^+^ T cell subsets and provided quantitative information on 185 kinases expressed in both CD4^+^ Tregs and Teffs. Most importantly, comparative kinome analyses revealed an aberrant expression of 11 kinases in Tregs, which substantiate the current hypothesis that TCR/CD28-mediated signaling cascades are altered in Tregs and further suggests that Tregs are affected in their cell-cycle progression. This knowledge might be of central importance for the identification of molecular targets for the manipulation of Treg numbers and function in various clinical settings.

## Materials and Methods

### Purification and Cultivation of Human T Cells

Isolation, characterization, and cultivation of human alloantigen-specific Teffs and Tregs have been described previously [23], [25]. Primary CD4^+^CD25^−^ Teffs and CD4^+^CD25^+^ Tregs were prepared from human peripheral blood of healthy donors by Ficoll gradient (BioChrom) centrifugation and MACS isolation using the CD4^+^CD25^+^ Regulatory T Cell Isolation Kit (Miltenyi Biotec). This study was approved by The Regional Ethics Review Board in Braunschweig. Peripheral blood was obtained with informed consent from healthy volunteers according to the Städtisches Klinikum Braunschweig gGmbH guidelines.

### T Cell Suppression Assays

T cell proliferation and suppressor activity were assessed by stimulating 3×10^4^ T cells in triplicates with irradiated LG2-EBV B cells and IL-2 in 96 flat-bottom microtiter plates (Nunc, Wiesbaden, Germany). At day 3 of the culture, cells were pulsed with 1 µCi/well of [H^3^]-thymidine for the final 16 h. T cell proliferation and suppressor activities were controlled prior each kinome experiment.

### Antibodies

For western blotting the following antibodies were used: anti-Actin (20–33 and beta-Actin, Sigma), anti-CDK2, anti-CDK6 (M2 and C-21, both Santa Cruz Biotechnology, Inc.), anti-Foxp3 (150D, BioLegend), anti-KCC2D/CaMKIIdelta (aa402–478, R&D Systems), anti-NEK9 (1F6, Abnova), anti-SRC (36D10, Cell Signaling Technology) and anti-STK4/Mst1 (EP1465Y, Epitomics). Flow cytometry was performed using APC-, FITC-, PE- and PerCP/Cy5.5-conjugated antibodies against CD4 (OKT4, eBioscience), CD25 (4E3, Miltenyi), CD127 (eBioRDR5, eBioscience) and Foxp3 (PCH101, eBioscience).

### Western Blotting

T cells were lysed in buffer containing 50 mM HEPES-NaOH pH 7.5, 150 mM NaCl, 1 mM EGTA, protease inhibitor cocktail (Complete, Roche) and 1% Triton-X100. Protein concentration was determined by Bradford protein assay (BioRad). Samples were separated by SDS-PAGE. Proteins were transferred to polyvinylidene fluoride membranes (Immunoblot™ PVDF membrane, BioRad). Membranes were probed with indicated primary antibodies and respective HRP-conjugated secondary antibodies (Dianova). Lumi-light western blotting solutions (Roche Diagnostics) were used as HRP substrates. Luminescence detection was performed using a CCD camera system (Fujifilm, Las3000, Raytest). AIDA densitometry software (version 4.15, Raytest) was used for signal quantification.

### Flow Cytometry

T cells were labeled using the indicated fluorochrome-conjugated antibodies (see above). Foxp3 staining was performed according to the manufacturer’s instructions (eBioscience). Acquisition was conducted on a FACSCanto™ II flow cytometry system (BD). Data were analyzed using FlowJo software (Tree Star, Inc.).

### Kinase Purification from T Cell Lysates

Teffs and Tregs were cultured as described above. For each kinome experiments 2×10^8^ resting Teffs and Tregs (one week post stimulation with EBV-transformed B cells) were used. T cells were IL-2-starved for 24 h und lysed in ice cold buffer containing 50 mM HEPES-NaOH pH 7.5, 1 M NaCl, 1 mM EGTA, protease inhibitor cocktail (Complete from Roche) and 1% Triton-X100. Lysates were centrifuged at 70.000 g for 30 min (4°C) and filtered using a 0.45 µm syringe membrane (Millipore). Protein concentrations of lysates were determined using Bradford protein assay (BioRad) to ensure appropriate loading of kinase affinity columns. For small molecule affinity chromatography (SMAC) the kinase inhibitors VI16743 and Purvalanol-B (Tocris) were used. Inhibitor synthesis and generation of kinase affinity matrix were conducted as described before [31], [33]. Columns (5/50 Tricorn, GE Healthcare) were equilibrated with buffer A (50 mM HEPES-NaOH pH 7.5, 1 M NaCl, 1 mM EGTA, 1 mM EDTA, 0.1% Triton-X100), loaded with T cell lysates (3 ml/h), washed with 60 column volumes of buffer A (6 ml/h) and equilibrated (2 h, 6 ml/h) with buffer B (50 mM HEPES-NaOH pH 7.5, 1 mM EGTA, 1 mM EDTA, 0.1% Triton-X100). Columns were eluted (6 ml/h) separately with 0.5% SDS at room temperature (RT). Eluates were pooled and concentrated using vacuum centrifugation. Proteins were extracted from eluate fraction by chloroform/methanol precipitation [88].

### Sample Preparation for Quantitative LC-MS/MS Analysis (iTRAQ™)

Protein dissolution, denaturation, tryptic digestion and quantitative peptide labeling were performed according to the manufacturer’s guidelines (Applied Biosystems). The following iTRAQ™ labels have been used: experiment 1 (Treg:114/Teff:117), experiment 2 (Treg:115/Teff:114), experiment 3 (Treg:116/Teff:114) and experiment 4 (Treg:117/Teff:114). iTRAQ™-labeled peptides generated from Teffs and Tregs were combined. Pooled samples were vacuum-dried and peptides were desalted using self-packed LiChroprep RP-18 (Merck) SPE columns. Desalted peptide mixture was further sub-fractioned by strong cation exchange chromatography (SCX). In brief, dried peptides were resolved and separated on a Mono S PC1.6/5 column (GE Healthcare) using an Ettan micro LC system (GE Healthcare). Peptide-containing fractions were vacuum-dried and desalted using RP-C18 µZipTip pipette tips (Millipore).

### LC-MS/MS Analysis and Database Search

LC-MS/MS analyses of SCX fractions were performed on an Acquity Ultra Performance LC system (Waters Corp.) connected to a Q-TOF micro (Waters Corp.) or LTQ Orbitrap XL device (Thermo Scientific). MS raw data were converted to data formats compatible with Mascot search engine (Matrix Science). Data from all SCX fractions were merged using Mascot Daemon (version 2.1.6). UniProt primary sequence database was used for protein identification (release 2011_03, with 525,997 entries; taxonomy *Homo sapiens* with 20,226 entries). In this study, proteins were only considered if they were identified at least with one unique peptide having an individual Mascot peptide score above 20, which indicated identity or extensive homology (p<0.05) using the following Mascot search parameters: enzyme, trypsin (specificity: K/R); maximum missed cleavages, 1; fixed modifications, iTRAQ 4-plex (K), iTRAQ (N-terminus), Carbamidomethyl or Methylthio (C); variable modifications, phosphorylation (S, T, Y), oxidation (M); MS/MS tolerance, 0,15 Da; peptide tolerance, 10 ppm, LTQ Orbitrap XL, 60 ppm, Q-TOF micro). False discovery rates (FDRs) were calculated using the software Scaffold (version Scaffold_3_00_06). On average protein FDRs less than 0,1% were determined. All MS-data associated with this manuscript are published in the PROteomics IDEntifications Database (PRIDE).

### Evaluation of Quantitative LC-MS/MS Data

Statistical evaluation of quantitative peptide data was conducted as described previously [40]. In brief, statistical evaluation was performed on the basis of Mascot result files (dat files) and was restricted to peptides being unique and unambiguously identified within the particular MS/MS data set (parameter setting: reporter mass delta, 0.02–0.05 Da; peptide cut off, respective significance boarder). iTRAQ reporter intensities were normalized. Likelihoods of relative peptide/protein abundances (here RF = Treg/Teff) were calculated using a MS device specific noise model (iTRAQassist). Resulting data were depicted as likelihood curves for every peptide/protein. Regulations were considered as significant by applying the following stringent criteria: (i) kinase regulation curves computed by the statistical noise algorithm iTRAQassist were clearly separated from the y-axis (RF = Tregs/Teffs = 1), (ii) the median of kinase regulation factors over all performed proteome experiments was 1.5>RF_median_>1.5 and (iii) kinases were differently expressed in two or more of the conducted experiments.

## Supporting Information

Figure S1 **Validation of iTRAQ-based MS/MS data by quantitative western blotting.** Graph shows the relative expression of the indicated kinases in *ex-vivo*-expanded Tregs (kinase expression in Teffs is set to 100%). Data shown represent (i) the mean of four independently conducted LC-MS/MS experiments (white bars, standard deviation is indicated by error bars) and (ii) western blot results obtained from a representative experiment (black bars).
(TIF)

Figure S2 **Reduced expression of CDK6 in freshly isolated Tregs.** CD4^+^CD25^−^ Teffs and CD4^+^CD25^+^ Tregs were freshly isolated from human peripheral blood of healthy donors **(A)** Purities of freshly isolated Tregs and Teffs were determined by flow cytometry using the indicated antibodies. Shown purities are representative for freshly isolated Tregs and Teffs analyzed in this study. **(B)** CDK6 expression is reduced in freshly isolated Tregs. Equal numbers of Tregs and Teffs were used for CDK6- and Actin-specific western blot analyses. One representative out of three experiments is shown. **(C)** Densitometric quantification of diminished CDK6 expression in Tregs. Actin protein levels were used for normalization. CDK6 expression in Teffs was set to 100%. Results from three individual donors are shown. Standard deviation is indicated by arrow bars.
(TIF)

Table S1 **The kinase complement of human Tregs and Teffs.** Kinases, which were identified in four independently performed LC-MS/MS experiments, are listed in alphabetic order by their UniProt names. ^1^UniProt name, ^2^Kinase family according to Manning et al. [22], ^3^Median Mascot Mowse score, ^4^Frequency of identification, ^5^Frequency of regulation (RF = Treg/Teff; −1.5<RF<1.5 (0); RF≤−1,5 (−); RF≥+1,5 (+); quantity of + and – is reflecting the number of experiments where the particular kinase showed a differential expression) and ^6^Median regulation over all conducted experiments.
(PDF)
