# Supplementary figures and images for: First Insight into the Kinome of Human Regulatory T Cells
Source: PLoS One. 2012 Jul 16;7(7):e40896. doi: 10.1371/journal.pone.0040896 (PMC3397934; doi:10.1371/journal.pone.0040896)

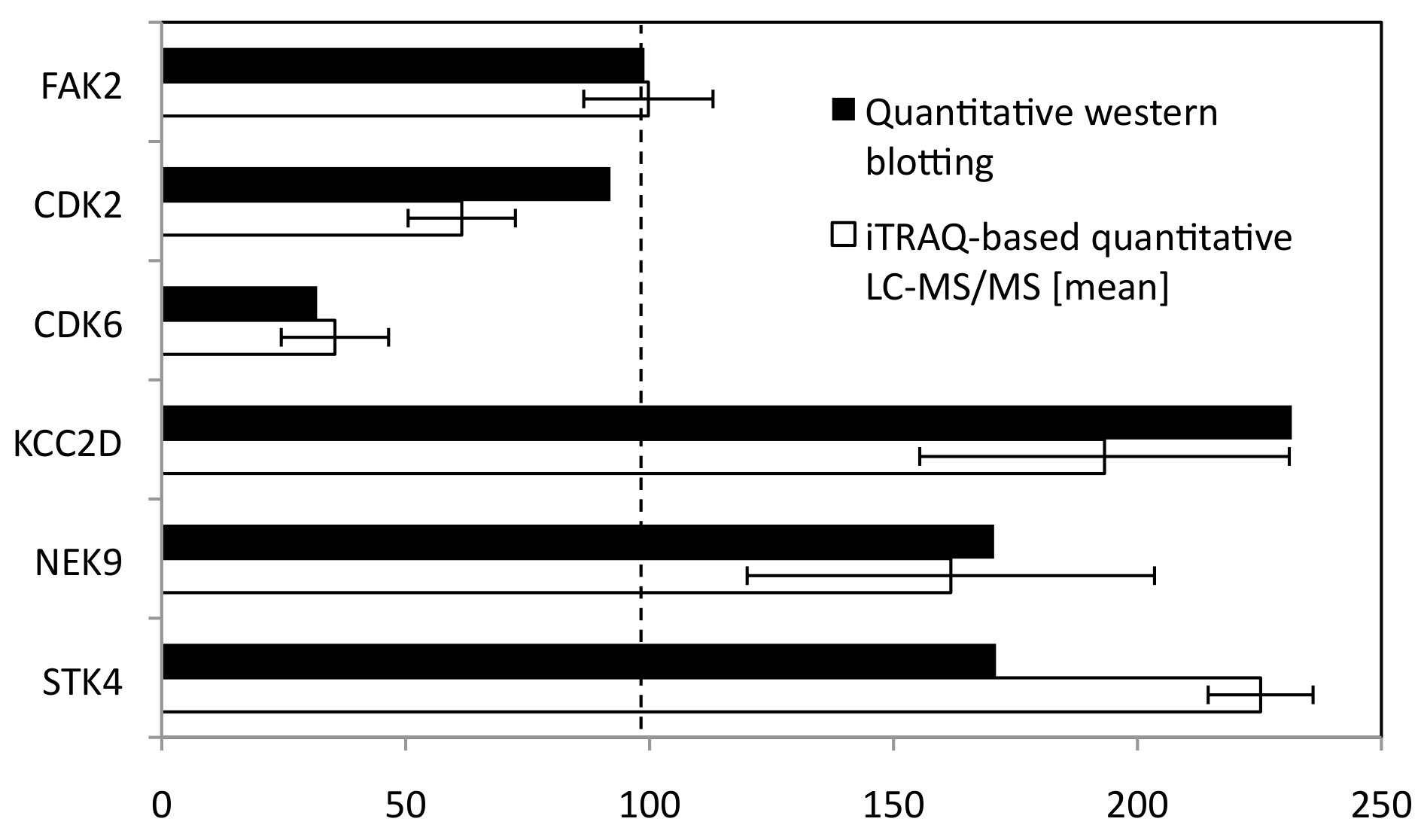

Supplement: Figure S1 — Validation of iTRAQ-based MS/MS data by quantitative western blotting. Graph shows the relative expression of the indicated kinases in ex-vivo-expanded Tregs (kinase expression in Teffs is set to 100%). Data shown represent (i) the mean of four independently conducted LC-MS/MS experiments (white bars, standard deviation is indicated by error bars) and (ii) western blot results obtained from a representative experiment (black bars). (TIF) [file pone.0040896.s001.tif]

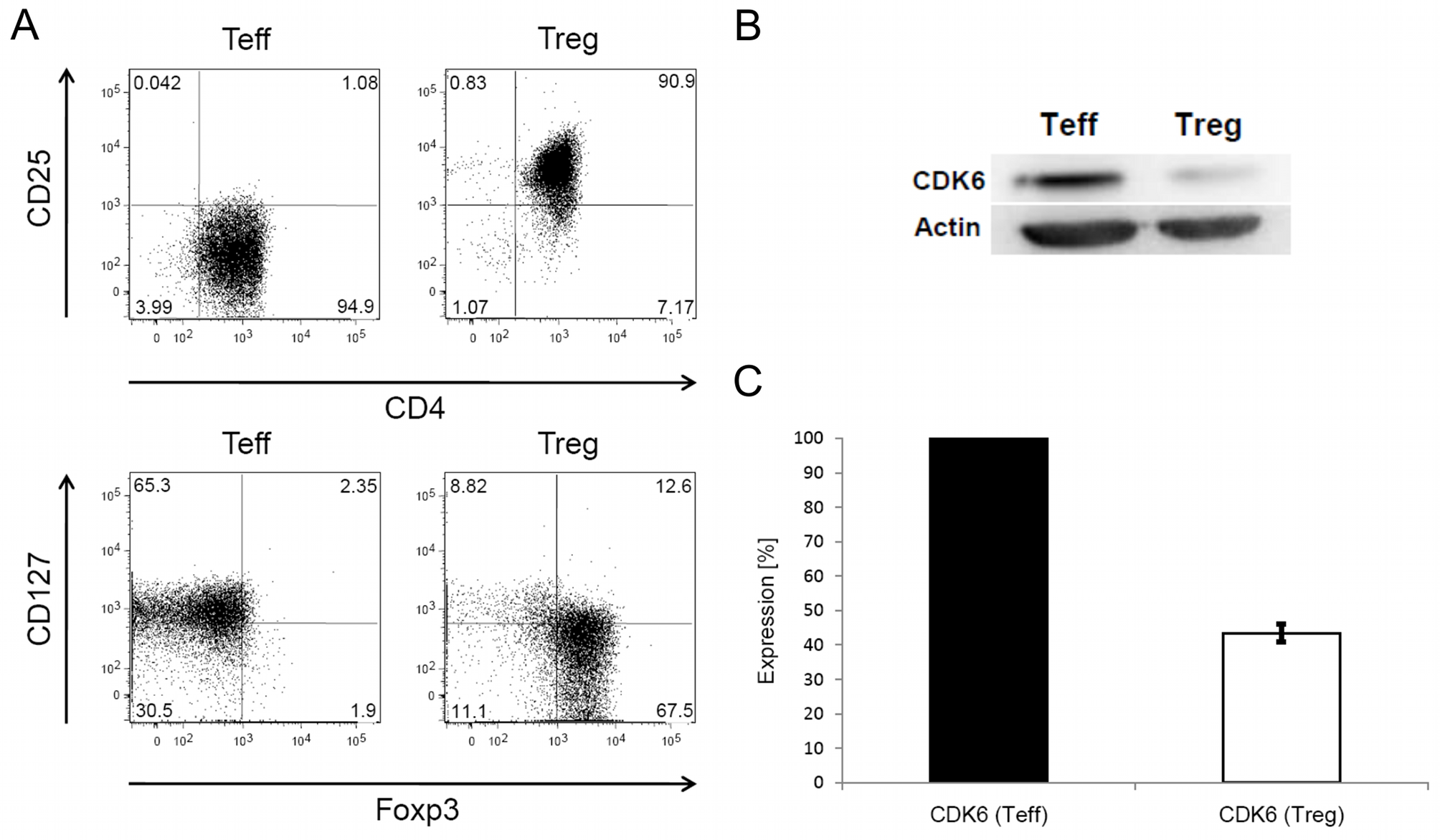

Supplement: Figure S2 — Reduced expression of CDK6 in freshly isolated Tregs. CD4+CD25− Teffs and CD4+CD25+ Tregs were freshly isolated from human peripheral blood of healthy donors (A) Purities of freshly isolated Tregs and Teffs were determined by flow cytometry using the indicated antibodies. Shown purities are representative for freshly isolated Tregs and Teffs analyzed in this study. (B) CDK6 expression is reduced in freshly isolated Tregs. Equal numbers of Tregs and Teffs were used for CDK6- and Actin-specific western blot analyses. One representative out of three experiments is shown. (C) Densitometric quantification of diminished CDK6 expression in Tregs. Actin protein levels were used for normalization. CDK6 expression in Teffs was set to 100%. Results from three individual donors are shown. Standard deviation is indicated by arrow bars. (TIF) [file pone.0040896.s002.tif]
