# Supplementary material for: First Insight into the Kinome of Human Regulatory T Cells
Source: PLoS One. 2012 Jul 16;7(7):e40896. doi: 10.1371/journal.pone.0040896 (PMC3397934; doi:10.1371/journal.pone.0040896)
Supplement: Table S1 — The kinase complement of human Tregs and Teffs. Kinases, which were identified in four independently performed LC-MS/MS experiments, are listed in alphabetic order by their UniProt names. 1UniProt name, 2Kinase family according to Manning et al. [22], 3Median Mascot Mowse score, 4Frequency of identification, 5Frequency of regulation (RF = Treg/Teff; −1.5<RF<1.5 (0); RF≤−1,5 (−); RF≥+1,5 (+); quantity of + and – is reflecting the number of experiments where the particular kinase showed a differential expression) and 6Median regulation over all conducted experiments. (PDF) [file pone.0040896.s003.pdf]

Table S1 – The kinase complement of human Tregs and Teffs.

Name[1] [UniProt]	Kinase family[2]	Mascot score[3] [median]	Frequency of identification[4]	Frequency of regulation[5]	Differential expression[6] [RF_{median}=Treg/Teff]
AAK1	Other	707	4	0	1,1
AAKB1	CAMK	162	1	0	-1,0
AAKB2	CAMK	236	1	-	-1,6
AAKG1	CAMK	396	1	0	-1,3
AAKG2	CAMK	80	1	0	-1,2
AAPK1	CAMK	1960	1	0	-1,2
ABL1	TK	96	1	0	-1,2
ABL2	TK	662	1	+	1,5
ACK1	TK	183	1	0	1,5
ACVR1	TKL	204	2	+	1,6
ALPK1	Atypical	32	1	0	1,2
ATM	Atypical	114	1	+	2,0
ATR	Atypical	21	1	0	-1,2
AURKB	Other	55	3	- -	-3,6
BMP2K	Other	1162	2	+	1,3
BMPR2	TKL	368	1	0	1,5
CD2L6	CMGC	108	1	0	1,0
CD2L7	CMGC	162	1	0	1,1
CDK1	CMGC	560	2	- -	-3,6
CDK10	CMGC	118	1	0	1,1
CDK2	CMGC	339	4	- -	-1,6
CDK3	CMGC	47	3	+++	2,0
CDK4	CMGC	72	3	0	1,2
CDK5	CMGC	421	4	++	1,4
CDK6	CMGC	474	4	- - - -	-3,2
CDK7	CMGC	1313	1	0	1,1
CDK9	CMGC	554	4	0	1,2
CDKL5	CMGC	30	1	0	1,4
CHKA	NPK	68	1	+	1,7
CHKB	NPK	148	1	+	1,6
CLK1	CMGC	292	1	0	1,1
CLK2	CMGC	435	1	-	-1,6
CLK3	CMGC	179	1	0	-1,0
CLK4	CMGC	150	1	+	1,5
CSK	TK	1846	2	+	1,6
CSK21	Other	578	4	+	1,3
CSK22	Other	664	4	0	1,2
CSK2B	Other	692	4	0	1,3
DAPK1	CAMK	35	1	0	1,5
DCLK3	CAMK	23	1	0	1,2
DGKA	NPK	21	1	+	2,2
DYR1A	CMGC	230	1	0	-1,1
DYR1B	CMGC	31	1	+	1,8
EF2K	Atypical	21	1	0	1,4
EPHA2	TK	105	1	0	1,1
EPHA4	TK	628	1	+	7,4
EPHB1	TK	79	2	++	6,9

EPHB4	TK	22	1	0	-1,2
ERN1	Other	1346	1	0	-1,4
FAK1	TK	130	1	0	1,3
FAK2	TK	1126	4	0	1,0
FER	TK	243	1	+	1,6
FES	TK	2804	1	-	-6,2
FGR	TK	195	1	0	1,2
FRAP	Atypical	95	1	0	1,2
FRK	TK	66	2	+	1,7
FYN	TK	1212	1	+	2,1
GAK	Other	113	3	0	-1,0
GRK6	AGC	31	1	+	2,0
GSK3A	CMGC	429	3	0	-1,2
GSK3B	CMGC	467	4	0	1,2
HIPK1	CMGC	69	3	0	-1,0
HUNK	CAMK	24	1	0	1,4
IGF1R	TK	101	1	0	1,1
IKKA	Other	166	1	-	-2,0
IKKB	Other	1859	1	-	-2,2
IKKE	Other	1596	2	0	1,2
ILK	TKL	368	1	0	1,3
IRAK3	TKL	265	1	0	1,2
IRAK4	TKL	32	1	0	-1,2
ITK	TK	79	4	+	1,3
JAK1	TK	2549	1	0	1,4
JAK3	TK	609	2	-	-1,4
KC1A	CK1	867	2	0	-1,1
KC1D	CK1	325	4	+	1,1
KC1E	CK1	566	2	0	-1,1
KC1G1	CK1	380	1	0	1,1
KC1G2	CK1	32	1	0	-1,0
KC1G3	CK1	272	1	0	1,0
KCC2B	CAMK	21	1	0	1,5
KCC2D	CAMK	885	4	++++	1,7
KCC2G	CAMK	364	3	+	1,2
KCC4	CAMK	43	1	-	-2,0
KCY	NPK	29	1	0	1,3
KPB1	NPK	48	1	0	1,1
KPB2	NPK	103	1	0	1,1
KPBB	NPK	176	1	0	-1,5
KPCA	AGC	317	1	-	-1,6
KPCB	AGC	89	1	0	1,1
KPCD2	CAMK	449	4	+	1,4
KPCD3	CAMK	104	1	0	-1,5
KPCL	AGC	208	1	0	-1,3
KPCT	AGC	287	2	0	-1,3
KPYM	NPK	677	2	+	1,8
KS6A1	AGC	1449	2	0	-1,2
KS6A3	AGC	3676	1	0	-1,3
KS6A4	AGC	243	1	0	1,1
KS6C1	AGC	25	1	0	1,4

KT3K	NPK	903	1	0	1,1
LCK	TK	84	3	+	1,3
LIMK1	TKL	979	1	0	-1,2
LIMK2	TKL	84	1	0	1,5
LYN	TK	1639	1	-	-2,2
M3K1	STE	568	1	0	1,2
M3K11	TKL	730	1	-	-1,6
M3K2	STE	647	2	0	1,0
M3K3	STE	798	1	0	-1,4
M3K5	STE	892	2	+	1,7
M4K1	STE	1599	1	0	1,3
M4K2	STE	248	1	0	1,1
M4K3	STE	414	1	0	1,1
M4K4	STE	955	1	0	1,2
MAPK5	CAMK	117	1	0	-1,2
MARK2	CAMK	1082	2	0	-1,1
MARK3	CAMK	778	1	0	1,1
MELK	CAMK	79	1	0	-1,5
MK01	CMGC	1282	4	-	-1,4
MK03	CMGC	865	2	-	-1,5
MK07	CMGC	41	1	-	-2,1
MK08	CMGC	205	3	-	1,3
MK09	CMGC	296	4	+	1,2
MK13	CMGC	30	1	0	1,5
MK14	CMGC	167	1	0	1,1
MK15	CMGC	210	1	-	-1,5
MLTK	TKL	1478	1	0	-1,1
MP2K1	STE	207	2	+	1,6
MP2K2	STE	62	1	0	-1,4
MP2K3	STE	208	1	0	1,1
MP2K6	STE	288	1	0	1,1
MRCKB	AGC	694	1	+	2,4
MUSK	TK	30	1	-	-2,5
MYLK	CAMK	52	3	0	-1,2
NAGK	NPK	39	1	0	1,3
NEK1	Other	254	1	+	2,2
NEK2	Other	57	1	-	-4,7
NEK5	Other	48	1	-	-4,7
NEK9	Other	1262	4	++	1,6
P4K2A	NPK	30	1	+	5,7
PAK4	STE	764	2	-	-1,4
PCTK1	CMGC	441	2	-	-1,1
PCTK2	CMGC	591	2	+	1,6
PDXK	NPK	888	4	+	1,2
PGK1	NPK	183	1	+	2,0
PHKG2	CAMK	109	1	0	-1,3
PI4KA	NPK	23	1	0	1,3
PK3CD	NPK	1197	2	0	-1,1
PLK1	Other	76	2	-	-1,2
PRKDC	Atypical	268	1	0	1,2
PRPK	Other	859	4	0	1,1

QSK	CAMK	34	1	0	-1,5
RIOK2	Atypical	384	1	0	1,3
ROCK1	AGC	234	2	0	-1,2
ROCK2	AGC	1175	1	0	1,1
ROR2	TK	35	1	0	1,2
SLK	STE	522	2	0	-1,1
SN1L2	CAMK	65	1	0	-1,4
SRC	TK	330	2	- -	-2,5
ST17B	CAMK	43	1	-	-1,5
STK10	STE	490	2	++	1,7
STK11	CAMK	99	1	0	1,2
STK16	Other	252	2	0	-1,1
STK24	STE	85	1	0	-1,0
STK3	STE	171	1	0	1,3
STK36	Other	83	1	0	-1,2
STK4	STE	519	4	++++	2,3
STK6	Other	187	2	-	-1,9
TAOK1	STE	571	1	0	1,2
TAOK2	STE	641	1	0	1,3
TAOK3	STE	314	1	0	-1,3
TBK1	Other	947	4	0	1,1
TEC	TK	159	1	-	-3,3
TGFR1	TKL	245	1	0	1,2
TGFR2	TKL	329	1	-	-1,9
TITIN	CAMK	113	1	0	1,4
TLK1	Other	34	1	0	1,4
TLK2	Other	262	1	0	1,0
TNIK	STE	247	1	0	1,3
TOPK	Other	86	1	0	-1,1
TSSK2	CAMK	31	1	0	-1,2
TXK	TK	96	1	0	1,1
TYK2	TK	410	1	0	-1,1
ULK3	Other	117	2	0	-1,0
VRK2	CK1	137	1	+	2,0
YES	TK	331	1	0	-1,2
YSK4	STE	29	1	0	1,1
